# Extirpation of the Inverted Uterus

**Published:** 1830

**Authors:** Willison Hughey

**Affiliations:** Pittsburgh


7. *Extirpation of the inverted uterus, by* Willison Hughey, M. D. *of Pittsburgh.*

In the third number of the *Baltimore Journal of Medicine and Surgery*, a case is related of the extirpation of this organ, under perhaps the only circumstances which can render the operation justifiable.

Dr. H. was called on the 6th of June to see a woman aged 39, wore down from a protracted illness of 10 years. She was at this time, represented to be laboring under profuse menorrhagia, and when he saw her, was so debilitated by the discharge that she fainted on the slightest exertion. The most active diffusible stimulants were conceived to be necessary, and were accordingly prescribed. On the next morning she was much better, and for the first time informed him that ten years before, a midwife, in rash attempts to remove the placenta, had inverted the uterus. For fifteen or twenty days after this she had been confined to her bed, from weakness and the inconvenience of the protrusion, but the organ slowly diminished in bulk, and she was able to get about, eventually with less inconvenience than might have been expected. During lactation the uterus remained free from disease, and after she weaned her child she menstruated regularly. In a short time, however, the organ enlarged and took on a diseased appearance.

When Dr. H. saw her it had, near the cervix, a pale red appearance without inflammation, but near the fundus it was highly inflamed, tense and unyielding, with several fissures and deep ragged ulcers interspersed, effusing a dark ichorous fluid, and very offensive. Near the fundus were several black spots, indicating a disposition to slough. For the next three days, in spite of the remedial measures, the disease advanced and the sloughing extended, giving rise to a renewal of the hemorrhage, when Dr. H. determined upon removing the entire organ.

Having placed my patient in the most advantageous situation, I carried a strong waxed ligature under the cervix uteri, brought the extremities together on the anterior surface, and cast a slack knot, so as to admit of its being moved up the vagina, on the included substance. I then took hold of the extremities of the ligature with my right hand, and with the left, passed my ligature as high as was possible on account of the inverted portion of the vagina. The ligature being placed where I intended it should remain, was tightened and secured with three knots ; this step in the operation occasioned exceeding pain ; so great was the torture which she suffered for twenty minutes, that I thought I shonld have been obliged to remove the cord, although I tied it as tight as possible. For a short time subsequent to the operation, I repented seriously the expedient which I had adopted ; although my patient had taken upwards of one hundred drops of laudanum, yet I was apprehensive that spasm would supervene and carry her off ; but fortunately the pain abated, and left her perfectly easy. The operation being performed, and the patient relieved from pain, I recommended quietness with a continuance of the tonic plan as before directed.

The patient, in six weeks after the operation, was sufficiently recovered to walk the distance of a mile, and soon recovered her health and spirits. More than a year subsequent she died of some other disease.—*Balt. Jour. of Medicine and Surgery.*

